# *Pseudomonas* sp. UW4 promotes garlic growth through systemic integration of auxin and ethylene pathways

**DOI:** 10.1186/s12870-026-08934-8

**Published:** 2026-05-13

**Authors:** Qizhang Wang, Jialu Zhao, Bernard R. Glick, Jie Tian

**Affiliations:** 1 Laboratory of Qinghai-Tibetan Plateau Germplasm Resources Research and Utilization, Academy of Agricultural and Forestry Sciences of Qinghai University, Xining, Qinghai Province 810000 China; 2 School of Life Sciences, Lanzhou University, Lanzhou, 730000 China; 3 Department of Biology, University of Waterloo, Waterloo, ON N2L 3G1 Canada

**Keywords:** Garlic (*Allium sativum* L.), Plant growth promoting bacteria (*Pseudomonas* sp. UW4), Transcriptome, Auxin, Ethylene

## Abstract

Plant growth-promoting rhizobacteria (PGPR) represent a sustainable strategy to enhance crop productivity, the multi-omics regulatory mechanisms underlying their growth-promoting effects in garlic remain poorly understood. In this study, we integrated physiological, phytohormone metabolomic, and transcriptomic analyses to systematically elucidate the growth-promoting mechanism of *Pseudomonas* sp. strain UW4 in garlic. Our results demonstrated that UW4 inoculation significantly improved aboveground morphological traits, including plant height, leaf length, leaf width, and pseudostem thickness, and optimized root system architecture by increasing total root length, root surface area, and root tip number. These morphological improvements were accompanied by enhanced photosynthetic pigment accumulation and increased biomass production. Phytohormone profiling revealed that UW4 inoculation elevated the levels of auxin (indole-3-acetic acid, IAA) and its precursors (L-tryptophan and tryptamine), while significantly reducing the content of 1-aminocyclopropane-1-carboxylic acid (ACC), the precursor of ethylene biosynthesis. Transcriptomic analysis identified 687 differentially expressed genes (DEGs), which were significantly enriched in auxin and ethylene signaling pathways. Mechanistically, UW4 upregulated the expression of *SAUR* (*Small auxin-up RNA*) to promote IAA synthesis, and suppressed the transcription of ethylene biosynthesis-related genes. Additionally, the downregulation of *PYL* (*Pyrabactin resistance 1-like*) genes indicated that UW4 also modulates the abscisic acid (ABA) signaling pathway. Quantitative real-time PCR (qRT-PCR) validation confirmed that the expression of *ACO* (*ACC oxidase*), a key rate-limiting enzyme in ethylene synthesis, was significantly downregulated in UW4 groups. Collectively, our findings demonstrate that UW4 optimizes garlic growth and yield potential by coordinately regulating auxin, ethylene, and ABA metabolism and gene expression, providing a theoretical foundation and elite microbial resource for the green and high-yield cultivation of garlic.

**Graphical Abstract:**

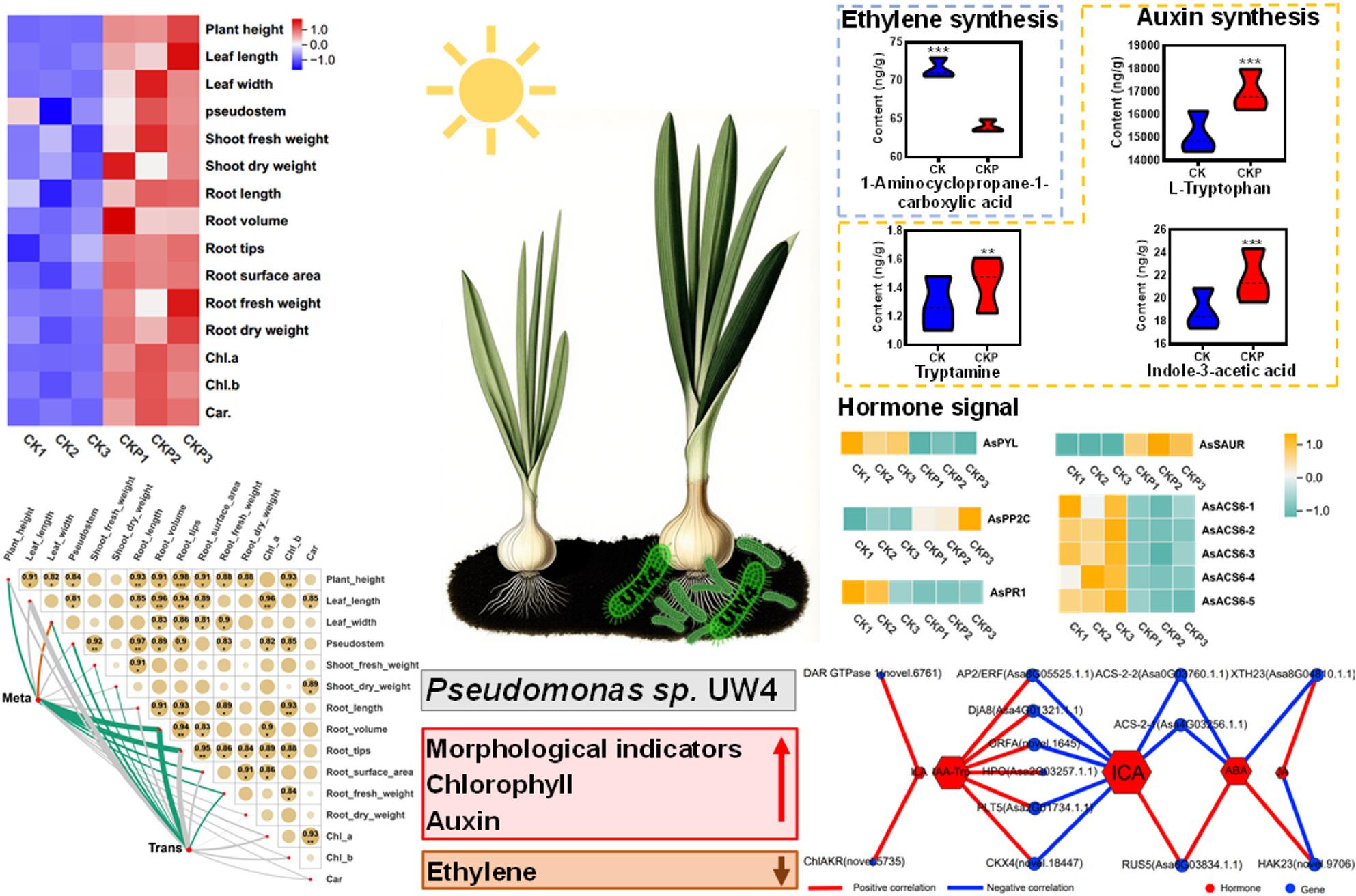

## Introduction

Garlic (*Allium sativum* L.), a bulbous plant, is a commercially significant crop valued worldwide for its culinary, nutritional, and medicinal properties. It belongs to the genus Allium, which comprises approximately 750 species, including seven that are widely cultivated across diverse agricultural regions [1]. Garlic is rich in bioactive compounds such as glycosides, vitamins, selenium, and allicin, and has been documented to exhibit a range of pharmacological activities, including antibacterial, anti-inflammatory, antihypertensive, and anticancer effects [2]. These attributes contribute to its status as a globally important medicinal and edible plant [3], China occupies a dominant position in global manufacturing, accounting for roughly 81% of the world’s dried garlic bulb output each year [4]. Garlic is grown under cool climatic conditions, which are critical for the formation of high-quality bulbs. Qinghai Province has emerged as a key cultivation area in China, with large bulbs, moderate pungency, high allicin content, and excellent sensory qualities [5]. Among these, microbial-based strategies application represents a promising approach to enhance yield and quality while reducing environmental impacts, thereby increasing the economic value of garlic production [6, 7].

Within the soil environment surrounding plant root systems, the microbial population in the rhizosphere greatly exceeds that in non-rhizosphere areas of the soil. This difference is mainly due to the richer and more abundant nutrients released by plant roots including amino acids, organic acids, and sugars which are essential for supporting microbial growth [8]. Current studies indicate that plant growth-promoting bacteria (PGPB) include more than 20 genera, among which *Pseudomonas*, *Bacillus*, and *Azotobacter* are prominent members. *Pseudomonas*, a Gram-negative aerobic bacterium, is one of the most widely used PGPB and grows stably between 25 °C and 30 °C [9]. It utilizes root exudates for its own growth and releases metabolites that help plants resist abiotic stress, thereby promoting plant growth, improving crop yield, and establishing a mutually beneficial relationship with the host plant [10]. Under stress conditions, plants typically increase ethylene production to plant inhibitory levels. However, many PGPB strains contain 1-aminocyclopropane carboxylate (ACC) deaminase enzyme, which can lower ethylene levels in plants allowing for a more balanced plant growth [11]. ACC is the direct precursor of ethylene and PGPB expressing ACC deaminase degrade ACC into α-ketobutyrate and ammonia, thereby reducing the synthesis of ethylene, which inhibits plant growth [12]. Under both abiotic and biotic stress, transcription of ACC synthase and ACC oxidase is enhanced, leading to increased ethylene synthesis. The presence of ACC deaminase-active PGPB can partially restrict this rise in ethylene [13]. As a result, bacterial ACC deaminase helps plants alleviate multiple stress responses, promote growth often reflected in increased root and shoot length, and improve adaptation. PGPB isolated from canola (*Brassica napus* subsp. *napus*) not only exhibit ACC deaminase activity but also enhance root growth and development, significantly raise average yield, and help prevent crop diseases through the secretion of indole-3-acetic acid (IAA) [14]. Moreover, IAA produced and secreted by PGPB can be absorbed by plant tissues, where it interacts with endogenous IAA. IAA may directly promote plant growth or induce the transcription of ACC synthase, which in turn stimulates ethylene synthesis [15, 16]. Elevated plant ethylene levels then feedback to inhibit IAA signaling, thereby restricting IAA-mediated growth. However, in the presence of ACC deaminase-producing PGPB, ethylene levels in plants are reduced, decreasing this feedback inhibition. Under such conditions, IAA signaling and plant growth can proceed without substantial ethylene accumulation. Therefore, ACC deaminase in PGPB helps lower ethylene levels, while IAA acts as a growth stimulating hormone [17]. Plant growth-promoting bacteria (PGPB) act as key regulators in sustainable agriculture and ecological remediation. In heavy metal-polluted soils, PGPB optimize rhizosphere microbial composition and network stability more efficiently, and synergize with indigenous beneficial taxa to enhance phytoremediation [18]. Under salt stress, Transgenic plants expressing either the UW4 gene (encoding ACC deaminase) or the *ACDS* gene driven by a root‑specific promoter hold promising potential for enhancing plant growth, seed yield and seed quality, especially on saline lands that are generally unsuitable for the cultivation of most crops [19]. *Pseudomonas sp.* UW4 secretes Flg22, which induces an immune response in the peripheral cells of wheat roots, colonises the wheat root tips, effectively controls root rot, and promotes wheat growth [20]. In drought regions, UW4 significantly promotes the growth of finger millet and effectively enhances its stress tolerance under arid conditions by increasing antioxidant enzyme activity, accumulating osmoregulatory substances and reducing oxidative damage [21]. These findings substantiate the profound potential of the UW4 agroecosystem to enhance plant fitness, while offering incisive perspectives into the mechanistic strategies underlying the unlocking of garlic’s growth potential within this study.

Progress in molecular biology has led to the broad application of multi-omics approaches including genomics, transcriptomics, proteomics, and metabolomics, in studying plant stress responses [22, 23]. For garlic, RNA-seq analysis has been employed to elucidate the regulatory mechanisms under low-temperature storage [24], and advanced our understanding of greening regulation during garlic processing [25]. Multi-omics studies have also elucidated the effective stress-alleviation strategies employed by halophytes under high-salinity stress through the accumulation of soluble organic compounds and flavonoids [26]. Integrated transcriptomics and metabolomics analyses have elucidated the mechanism by which *C. glutamicum* strain TR26 increases tryptophan production through nitrogen deprivation, providing new insights into microbial biosynthesis [27]. Metabolomics analysis of plant–microbe interactions has revealed that a metabolically symbiotic relationship exists between *Bacillus subtilis* SQR9 and *Pseudomonas syringae* XL272, which synergistically enhances their beneficial effects on plants [28]. Researchers also utilised metabolomics to discover that *Phytophthora capsici* LT263 supplements phenylalanine metabolism and enhances salicylic acid (SA) signal by producing phenylpyruvic acid, revealing previously unrecognised immune components within the metabolic network [29]. Similarly, it was found that *Bacillus megaterium* NCT-2 improves nutrient uptake primarily by enhancing soil nitrate reductase activity; transcriptomic and non-targeted metabolomic analyses confirmed the key roles of phenylalanine biosynthesis and tryptophan metabolism in this process [30]. Integrated transcriptomic and metabolomic approaches have been widely used to dissect plant-microbe interactions and regulatory networks. Multi-omics data provide powerful insights into how plant growth‑promoting rhizobacteria enhance stress tolerance and nutrient use efficiency through metabolic crosstalk and signal regulation, supporting the development of agricultural microbial inoculants. This study combined transcriptomics and metabolomics to reveal gene and metabolic changes in garlic responding to rhizobacteria, and clarified the growth‑promoting mechanisms, providing a theoretical basis for efficient garlic cultivation.

## Materials and methods

### Growth of bacterial strains and inoculum

*Pseudomonas* sp. UW4, a plant growth-promoting bacterium originally isolated from the rhizosphere of reeds growing on the campus of the University of Waterloo, was cultured in Luria-Bertani (LB) medium in sterile tubes [31]. This bacterium produces both IAA and ACC deaminase. The cultures were incubated at 30℃ for 24 h with orbital shaking at 220 rpm. Bacterial cells were harvested by centrifugation at 4000 rpm for 10 min and washed twice with sterile distilled water. The pellet was then resuspended in sterile distilled water, and the bacterial suspension was adjusted to a concentration of 1.0 × 10⁶ colony-forming units per milliliter (CFU/mL) for use as the inoculant.

### Plant growth conditions and inoculation

Garlic (*Allium sativum* L.) originates from cultivated varieties at the Horticultural Institute of the Qinghai Academy of Agricultural and Forestry Sciences, constituting a distinctive local crop of Qinghai Province. Cloves were surface-disinfected in 70% ethanol for one minute, followed by three rinses with sterile distilled water (3 min per rinse). The cloves were then planted in pots filled with sterile vermiculite and placed in an illuminated growth chamber set at 25 °C, with a 14/10 h light/dark cycle and 60% relative humidity, for germination and seedling growth over 7 days. Uniform seedlings were aseptically transplanted into larger pots (15 cm diameter × 15 cm height) containing 300 g of sterile dry substrate (BaseSubstrate#413, Klasmann-Deilmann GmbH, Germany Ltd.) and grown for another 3 days under the same conditions. Control group (CK) initially received 200 mL of distilled water, followed by 100 mL every 5 days to maintain soil moisture. Within the UW4 strain treatment group (CKP), plants were initially irrigated with 200 ml *Pseudomonas* sp. UW4 suspension (1.0 × 10⁶ CFU/ml). During subsequent growth, no further irrigation with UW4 suspension was applied. Later cultivation procedures were consistent with CK, with subsequent irrigation of 100 ml of distilled water every 5 days. After 20 days of treatment, three biological replicates were randomly selected from each group for morphological measurements and analysis.

### Growth parameters measurement

On day 20 of treatment, five uniform plants per treatment were selected for assessment. Plant height, leaf length, and leaf width were measured using a ruler, pseudostem diameter was recorded with dial calipers, photosynthetic pigments were measured according to Arono [32]. Shoot and root fresh weights were determined by analytical balance. Root indexes were analyzed by scanning (WinRHIZO™ software, Regent Instruments) to quantify total root length, root surface area, average root diameter, total root volume, root tips and root projected area. Subsequently, samples were oven-dried at 105℃ for 30 min, then at 70℃ to constant weight.

### Plant hormone detection

#### Plant hormone extraction

Take three biological replicates separately, and then commission the company to carry out the subsequent extraction process. HPLC-grade acetonitrile, methanol, acetic acid, formic acid, Milli-Q water, and all standards were purchased from commercial sources; standard stock solutions (1 mg/mL in MeOH) were stored at -20 °C and diluted to working solutions before analysis. Fresh plant samples were harvested, frozen in liquid nitrogen, ground to powder, and 50 mg aliquots were extracted with methanol/water/formic acid (15:4:1, V/V/V) with internal standards added; after vortexing, centrifugation, evaporation to dryness, reconstitution in 80% methanol, and filtration through a 0.22 μm membrane, extracts were analyzed by UPLC-ESI-MS/MS (UPLC: ExionLC™ AD; MS: Applied Biosystems 6500 Triple Quadrupole) on a Waters ACQUITY UPLC HSS T3 C18 column with a gradient elution program (5%-95% acetonitrile containing 0.04% acetic acid, 0–12 min), flow rate 0.35 mL/min, column temperature 40 °C, and injection volume 2 µL. Mass spectrometry was performed on a QTRAP^®^ 6500 + system in positive/negative ion modes, with ESI parameters set as source temperature 550 °C, ion spray voltage 5500 V/-4500 V, and curtain gas 35 psi; phytohormones were quantified via scheduled MRM, with data acquired and analyzed using Analyst 1.6.3 and Multiquant 3.0.3 software, respectively, and DP and CE optimized for each MRM transition (https://www.metware.cn/).

#### Data analysis

To systematically analyze metabolomic data, the following steps were performed in sequence: Initially, hierarchical cluster analysis (HCA) and Pearson correlation coefficients (PCC) were conducted using the R package pheatmap. HCA results for samples and metabolites were visualized as heatmaps with dendrograms, where normalized metabolite signal intensities (after unit variance scaling) were displayed as a color spectrum; concurrently, PCC between samples were calculated via the cor function in R and presented as heatmaps alone. Next, significantly regulated metabolites between groups were identified based on t-test P-value and absolute Log2 fold change (Log2FC). Subsequently, the identified metabolites were annotated using the KEGG COMPOUND database (http://www.kegg.jp/kegg/compound/) and then mapped to the KEGG PATHWAY database (http://www.kegg.jp/kegg/pathway.html). Ultimately, metabolite sets enrichment analysis (MSEA) was performed on pathways mapped with significantly regulated metabolites, and the significance of these pathways was determined by the p-value of the hypergeometric test. (https://www.metware.cn/).

### RNA extraction, sequencing and data analysis

#### RNA extraction, sequencing

Total RNA from JQ-00106 (LG2) raw seedling leaf, was extracted using a TRIzol kit (Thermo Fisher Scientific, USA). Measurement of the quality and concentration of RNA was performed using a One Drop OD-1000 spectrophotometer (One Drop, OD-1000, China). Quality control of the sequencing data indicates that the Q20 value is greater than 97%, the Q30 value is greater than 93%, and the GC content is greater than 44%. Raw sequencing reads were processed for quality control and adapter trimming using fastp. Clean high-quality reads were then used for de novo transcriptome assembly with Trinity, followed by transcript clustering and redundancy removal using Corset. For functional annotation, nucleotide sequences were aligned against public databases, and putative protein sequences were annotated against the Pfam database using HMMER. Transcript abundance was quantified as FPKM with RSEM. Differential expression analysis was performed using DESeq2 for replicated experiments and edge R for non-replicated comparisons. Genes with adjusted p-values (Benjamini-Hochberg correction) and absolute log₂ fold-change values exceeding the threshold were identified as differentially expressed genes (DEGs). Functional enrichment analyses of DEGs, including GO terms and KEGG pathways, were conducted based on the hypergeometric distribution using the Metware online platform (https://www.metware.cn/).

#### Data analysis

Raw sequencing data were first quality-filtered using fastp v0.19.3 to remove adapter sequences, as well as paired reads containing more than 10% ambiguous bases (N) or over 50% low-quality bases (Q < 20), and all downstream analyses were based on the obtained clean reads. Novel transcripts were then assembled de novo with String Tie v1.3.4d, which employs a network-flow algorithm and exhibits superior completeness, accuracy and efficiency relative to Cufflinks. Gene expression levels were subsequently quantified as FPKM values based on read counts calculated by feature Counts v1.6.2. Finally, differential expression analysis between groups was performed using DESeq2 v1.22.1, with P-values adjusted by the Benjamini–Hochberg approach, and significantly differentially expressed genes were identified using adjusted P-values and |log₂ fold change| as thresholds (https://www.metware.cn/).

### qRT PCR analysis

Leaf and root samples of garlic were harvested at 0 h, 4 h, 12 h, 1 d, 2 d, and 3 d following the initiation of treatments. Total RNA was extracted from frozen tissues using TRNzol reagent according to manufacturer protocol. RNA integrity was examined by 1% agarose gel electrophoresis, and RNA concentration and purity were assessed using a TGem micro-spectrophotometer. First-strand cDNA was synthesized from 1 µg of total RNA using the Fast Quant First Strand cDNA Synthesis Kit following the recommended procedure. Quantitative real-time PCR (qRT-PCR) was performed for the reference gene and *ACO* (F: 5’-ATCATTCCATTGTTGT-3’, R: 3’-TCTGTTTTTCCTCTTC-5’) in a 20 µL reaction mixture consisting of 1 µL cDNA (100 ng/µL), 0.4 µL each of forward and reverse primers (10 µmol/L), 10.0 µL 2× SYBR Green qRT‑PCR Master Mix, and nuclease-free dd H₂O. All reactions were carried out in three technical replicates. After amplification, melting curve analysis was performed to verify the specificity of the amplified products. Relative expression levels of the target gene were calculated using the 2⁻^ΔΔCt^ method.

### Statistical analysis and mapping

*Allium sativum* root morphology data were automatically calculated using the WinRHIZO™ software (Regent Instruments Inc., WinRHIZO STD4800 LA2400). Leaf morphological indices and physiological data underwent statistical analysis with Microsoft Excel 2010 and graphical representation with GraphPad Prism software (version 8.0). Data significance was assessed using the T-test.

## Results

### Garlic above ground morphological growth indicators and root index

**Fig. 1 Garlic morphological indicators and biomass:**
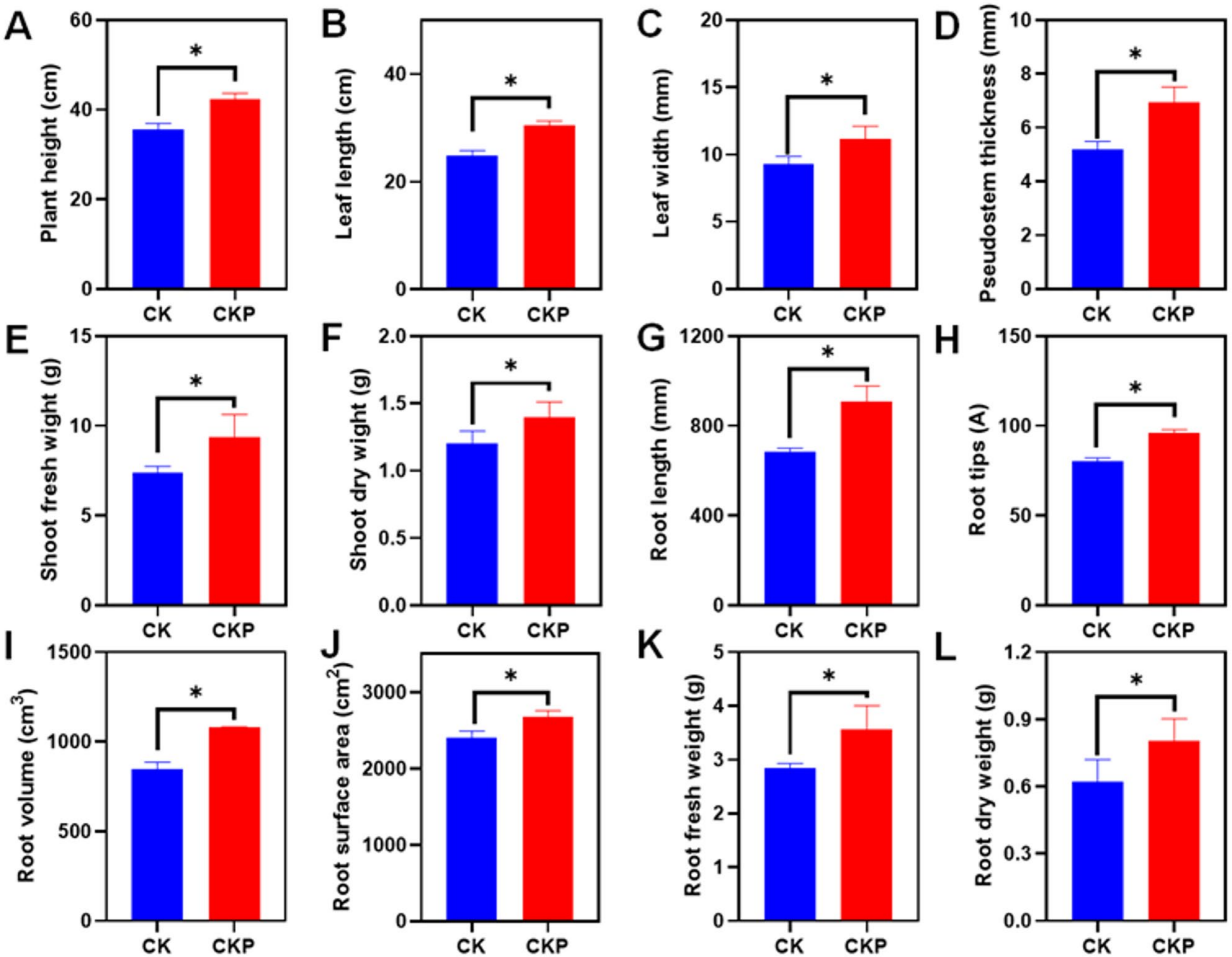
Note: CK represents the untreated control group, and CKP represents the UW4-inoculated treatment group. The panels show: (**A**) plant height, (**B**) leaf length, (**C**) leaf width, (**D**) pseudostem thickness, (**E**) shoot fresh weight, (**F**) shoot dry weight, (**G**) root length, (**H**) root tip number, (**I**) root volume, (**J**) root surface area, (**K**) root fresh weight, and (**L**) root dry weight. Data are presented as means ± standard error (SE). Asterisks indicate significant differences between the CK and CKP groups (P < 0.05, t-test). Three biological replicates per treatment, same as below

Plant height, leaf length, leaf width and pseudostem are morphological indicators that directly reflect a plant degree of adaptation to the external environment. Compared with control plants (CK), UW4 treatment (CKP) increased garlic plant height (Fig. 1-A), leaf length (Fig. 1-B), leaf width (Fig.1-C) and pseudostem (Fig.1-D) by 15.91%, 18.25%, 16.92% and 25.02%, respectively. Among these morphological indicators, UW4 strain showed the strongest effect in promoting pseudostem. Evaluating the yield-enhancing effect of this growth-promoting bacterium by quantifying fresh and dry plant weights revealed that UW4 strain significantly increased both the fresh and dry weights of the aboveground parts, with increases of 20.80% and 13.57% respectively. UW4 also significantly promoted garlic root growth. After 20 days of treatment, compared with the control group (CK), the total root length (24.21%) (Fig. 1-G), root surface area (10.40%) (Fig.1-H), number of root tips (16.95%) (Fig. 1-I) and root volume (21.36%) (Fig. 1-J) of garlic treated with UW4 all increased. UW4 treatment significantly promoted both garlic root fresh and dry weights, with increases of 20.45% and 22.76% respectively (Fig. 1-K, Fig. 1-L).

### Chlorophyll contents and comprehensive analysis

**Fig. 2:**
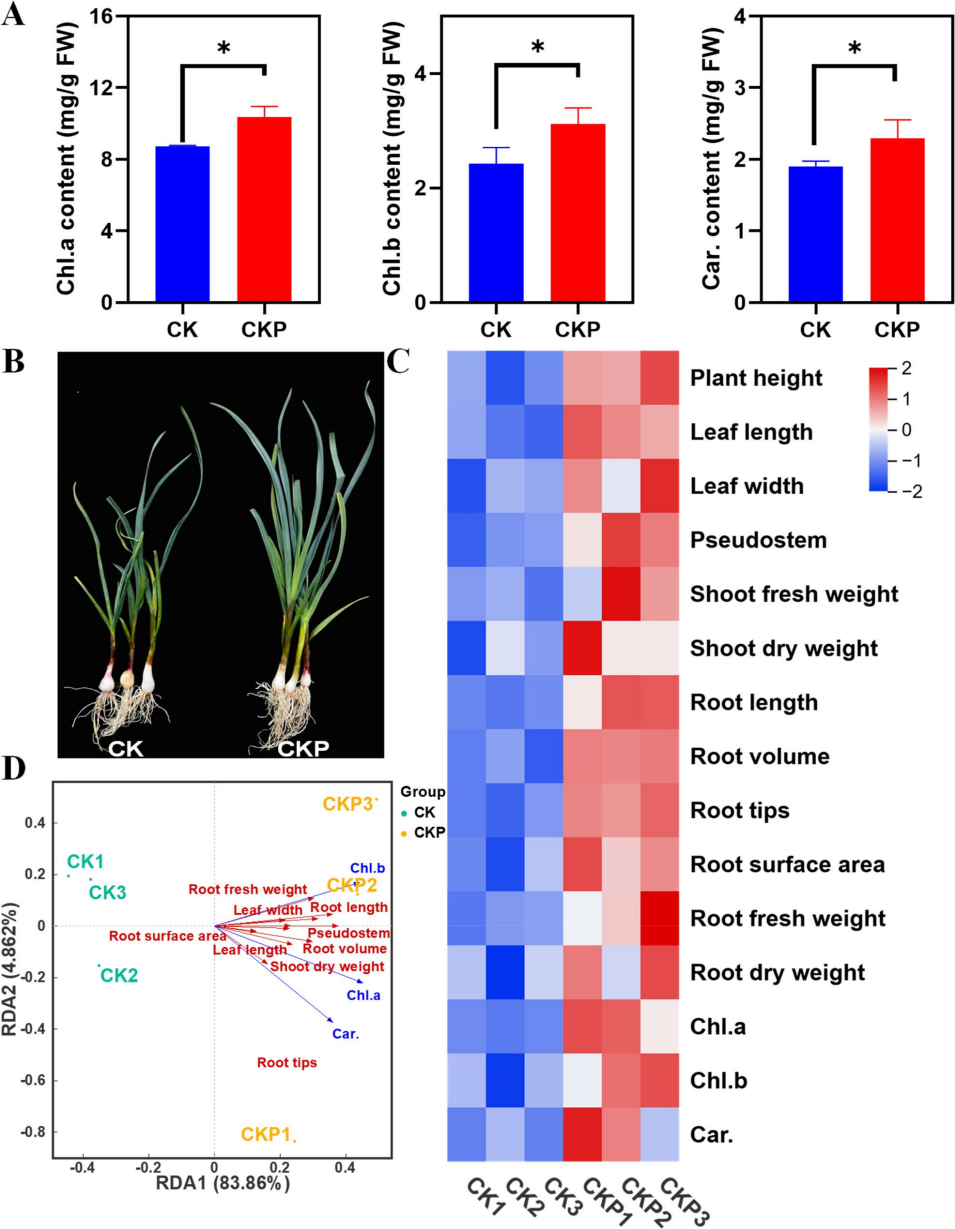
Garlic photosynthetic pigment content and comprehensive analysis. Note: (**A**) The figure shows the content of chlorophyll a (Chl.a), chlorophyll b (Chl.b), and carotenoids (Car.). Three biological replicates per treatment (t-test). **B** shows plant morphology under control groups and strain UW4-treated groups treatment. **C** Heatmaps illustrate visible differences among garlic morphological and physiological indicators. CK1, CK2, CK3 represent different control groups; CKP1, CKP2, CKP3 represent different strain UW4-treated groups, same as below. **D** Redundancy Analysis demonstrated characteristic indicators across different groups

Chlorophyll is a vital plant component enabling plants to perform photosynthesis, converting carbon dioxide into organic matter while releasing oxygen through the utilization of light energy. After 20 days of strain UW4 treatment, the contents of chlorophyll a, chlorophyll b, and carotenoids reached 10.38 mg/g FW, 3.11 mg/g FW, and 2.29 mg/g FW, respectively. Compared to normal growth (without strain UW4), strain UW4 application resulted in increases of 16.12%, 22.1%, and 17.16% in chlorophyll a, chlorophyll b, and carotenoid content, respectively (Fig. 2-A). After inoculating garlic roots with strain UW4, growth performance was significantly superior to the growth performance of the non-inoculated control plants, exhibiting more developed root systems and robust above-ground parts (Fig. 2-B). Further Heatmap analysis of indicator changes across treatments revealed an upward trend in all above-ground and root indicators following strain UW4 inoculation (Fig. 2-C). Redundancy analysis (RDA) of growth and physiological indicators across two treatments revealed that the RDA1 and RDA2 axes accounted for 88.762% of the total information content. In Fig. 2-D, CK and CKP are positioned on opposite sides of the axes, emphasizing the fact that that strain UW4 significantly promotes plant growth. Comparison of loadings confirms that strain UW4 most markedly enhances chlorophyll a, chlorophyll b, and carotenoids, garlic leaf length, leaf width, root length, fresh aboveground weight, and dry aboveground weight.

### Transcriptome differential gene statistics and screening

**Fig. 3:**
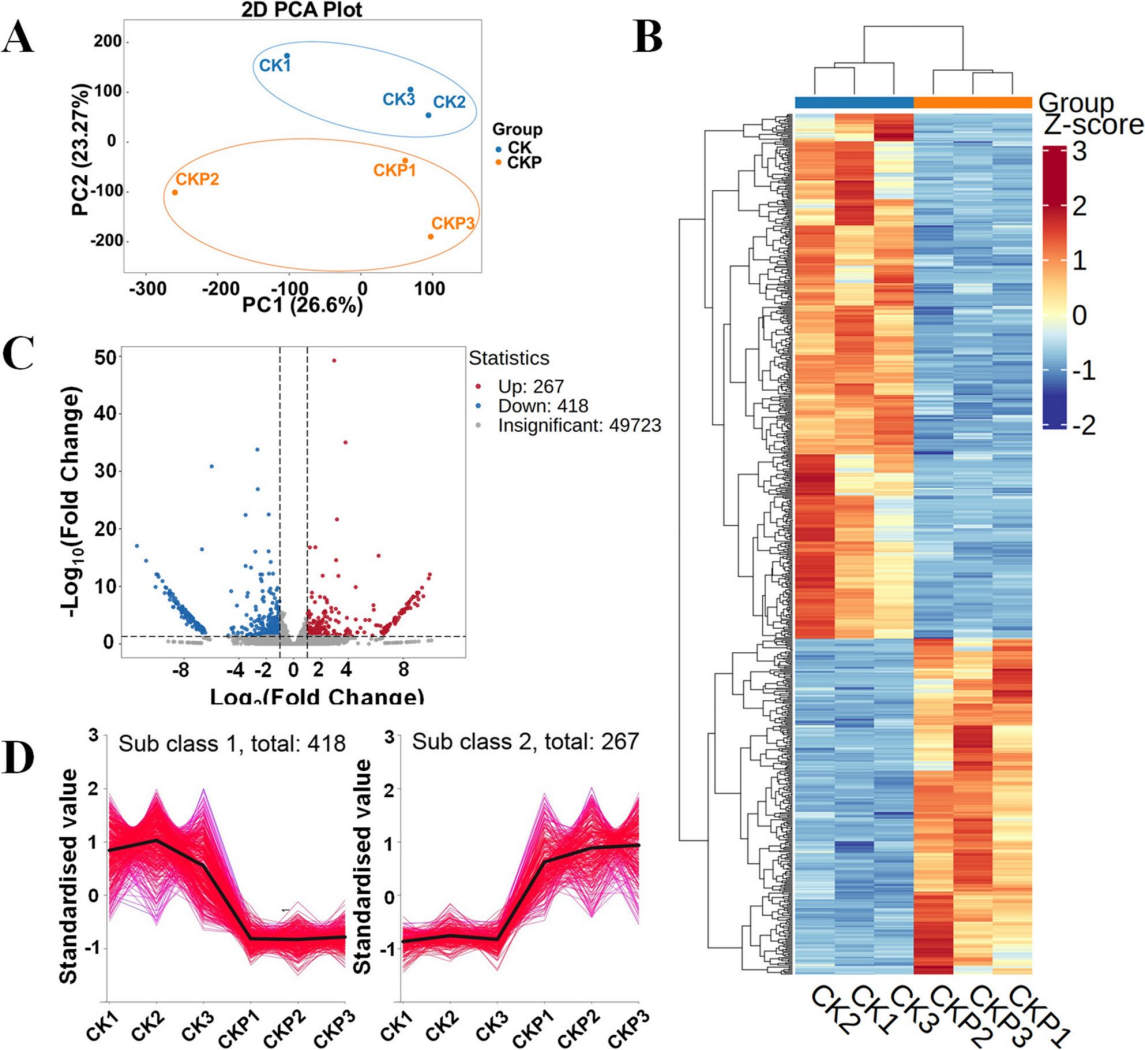
Garlic Transcriptome Differentially Expressed Genes. Note: (**A**) Different treatment principal component plots. **B** Expression heatmap analysis of differentially expressed genes. **C** Differentially expressed gene volcano plot. **D** K-means clustering analysis of differentially expressed genes

Integral relationships among samples were assessed to validate the experimental design and identify potential confounding factors. PCA analysis revealed that the first two principal components, PC1 and PC2, explained 26.6% and 23.27% of the total variance, respectively. As shown in Fig. 3-A, samples from the experimental group CKP and the control group CK formed two distinct clusters along the PC1 axis, indicating that the strain UW4 treatment was the primary driver of transcriptomic variation. reflecting high reproducibility of experimental procedures. The heatmap of differentially expressed genes (Fig. 3-B) visually corroborated the PCA findings, clearly demonstrating that these genes distinguish experimental from control samples and revealing potential co-expression patterns among differentially regulated genes. This study screened 50,408 genes, identifying 685 differentially expressed genes. Volcano plots and K-means analysis further identified 267 upregulated genes and 418 downregulated genes (Fig. 3-C and -D).

### Phytohormone signaling pathway analysis

**Fig. 4:**
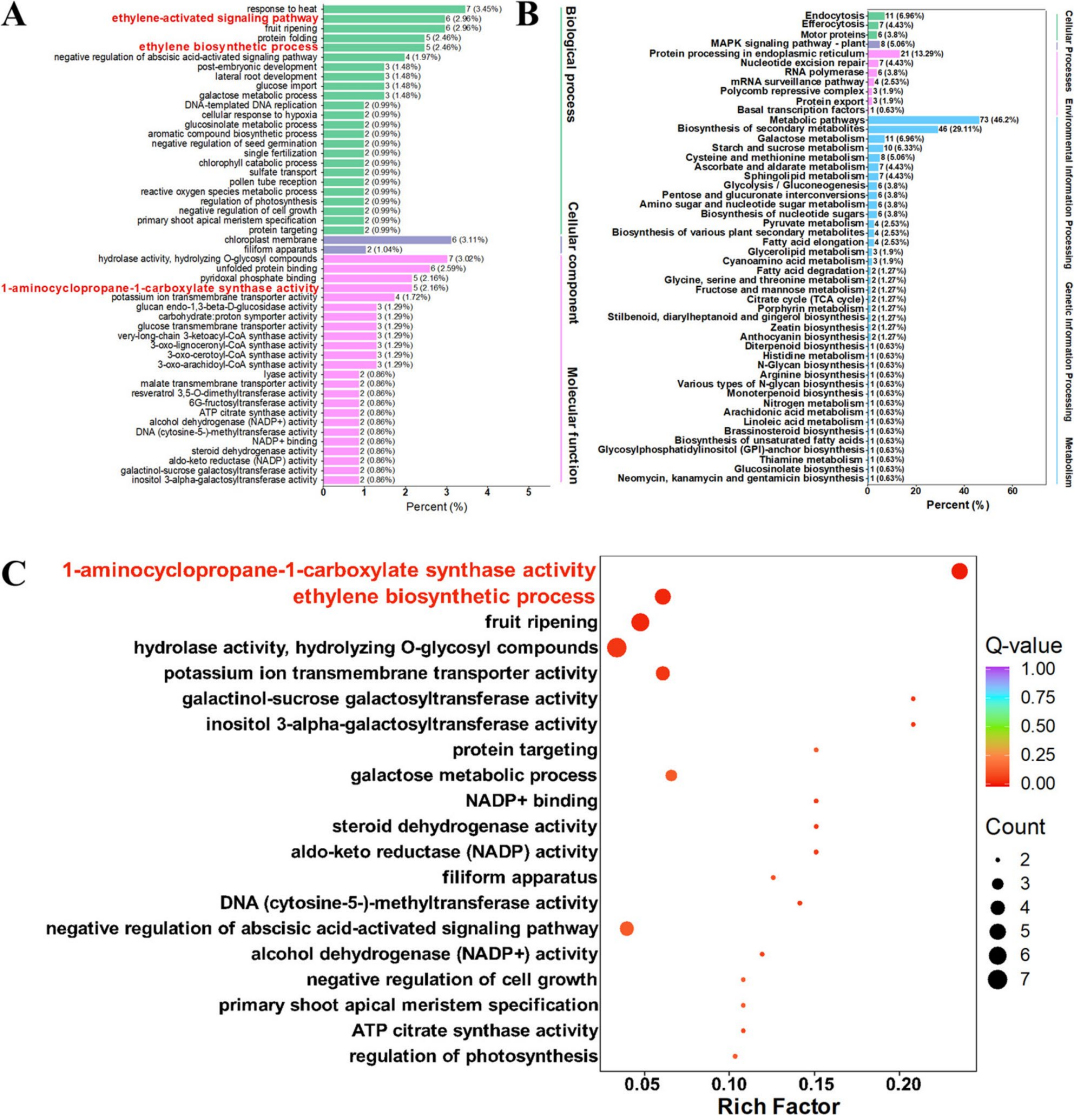
Enrichment analysis of key differentially transcribed genes. Note: (**A**) GO enrichment analysis, categorized into biological process (green), cellular component (blue), and molecular function (purple). Ethylene biosynthesis-related terms are highlighted in red. **B** KEGG pathway enrichment analysis, grouped by functional categories. The bar length represents the proportion of enriched genes. **C** GO enrichment bubble plot. The x-axis represents the Rich Factor, bubble color indicates the Q-value, and bubble size represents the number of enriched genes

GO (Gene Ontology) enrichment analysis (Fig. 4A-B) indicates that differentially expressed genes are primarily concentrated in the biological process and molecular function categories. Biological process enrichment is mainly observed in ethylene-activated signaling pathway, ethylene biosynthetic process, and negative regulation of the abscisic acid activated signaling pathway. Molecular function enrichment primarily targeted 1-aminocyclopropane-1-carboxylate synthase activity, hydrolase activity, hydrolyzing O-glycosyl compounds, and unfolded protein binding. KEGG pathway analysis (Fig. 4C) revealed that differentially expressed genes were enriched in cellular processes, environmental information processing, genetic information processing, and metabolism. Metabolism showed the highest enrichment of differentially expressed genes, primarily in Metabolic pathways and Biosynthesis of secondary metabolites. This strongly suggests that strain UW4 modulates plant hormone stress responses through gene expression levels related to metabolism and secondary metabolite biosynthesis. Combined with GO enrichment analysis, UW4 regulates these gene expression changes to activate plant hormone stress responses, primarily modulating ethylene and abscisic acid signaling to facilitate garlic growth.

**Fig. 5:**
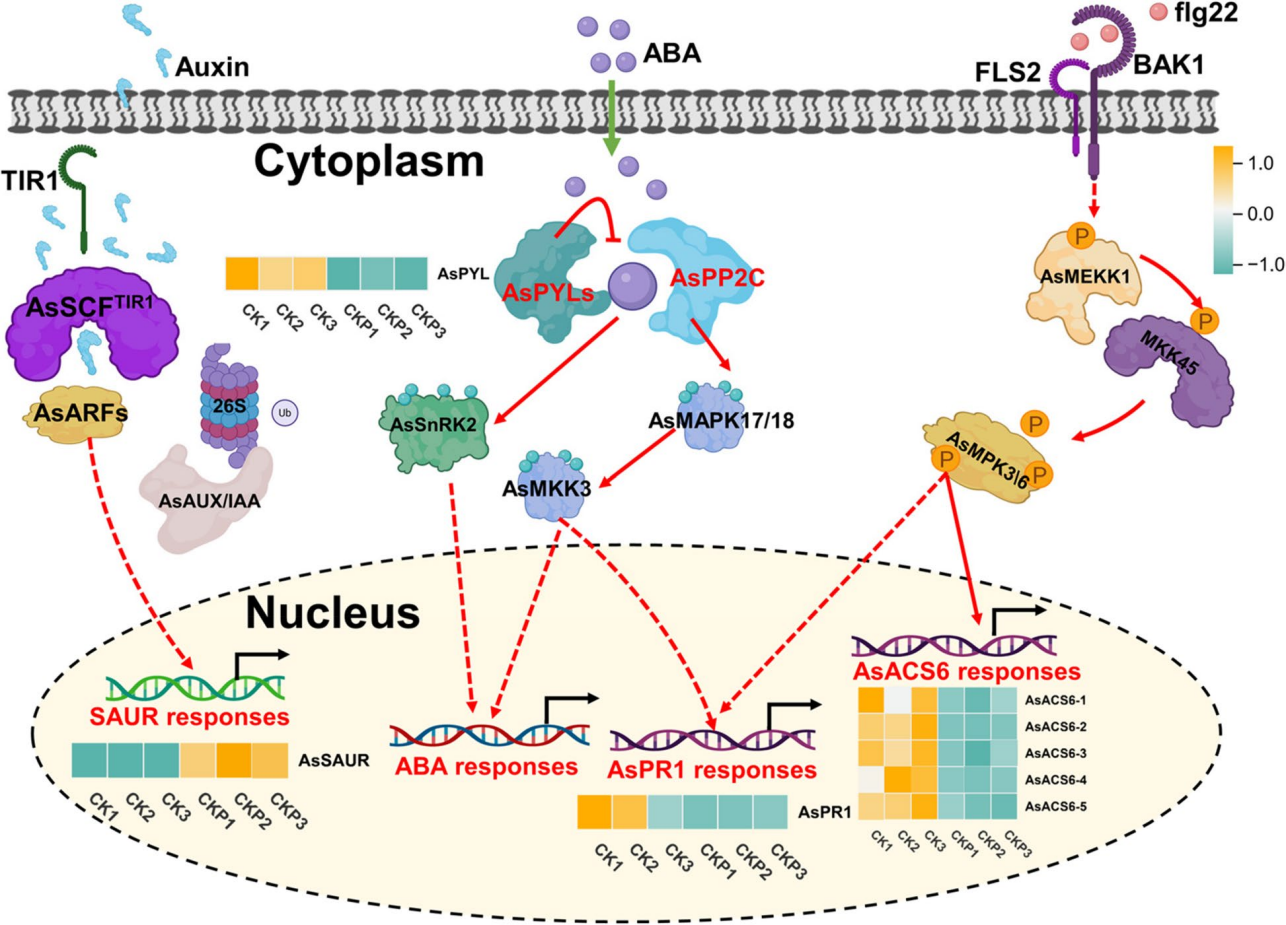
Auxin and ethylene pathways. Note: Auxin is perceived by *TIR1*, leading to *AsAUX/IAA* degradation via the *AsSCF/TIR1* complex, which releases *AsARFs* to activate *AsSAUR*. ABA binds *AsPYLs* to inhibit AsPP2C, activating AsSnRK2 and downstream *AsMAPK3/6* to regulate *AsPR1*. *AsMKKs-AsMPK3/6* cascade induces AsACS6. Heatmaps display gene expression under CK/CKP treatments (blue to yellow: low to high expression)

KEGG pathway enrichment analysis revealed that differentially expressed genes were predominantly enriched in the auxin and ABA (Abscisic acid) signaling pathways, and were also involved in modulating the expression of genes associated with ethylene biosynthesis and the MAPK (Mitogen-activated protein kinase) cascade. The UW4 strain may promote garlic growth by inducing the expression of *SAUR* family genes to alter endogenous auxin levels; in the ethylene signaling pathway, this strain mainly inhibits the expression of garlic *AsACSs* genes, thereby reducing the rate of ethylene biosynthesis and decreasing endogenous ethylene accumulation. Meanwhile, UW4 represses the expression of the *PYL* gene in garlic and downregulates the downstream target gene *AsPR1* (*Pathogenesis-Related proteins 1*) via the MAPK cascade (Fig. 5).

### Key differences plant hormone screening

**Fig. 6:**
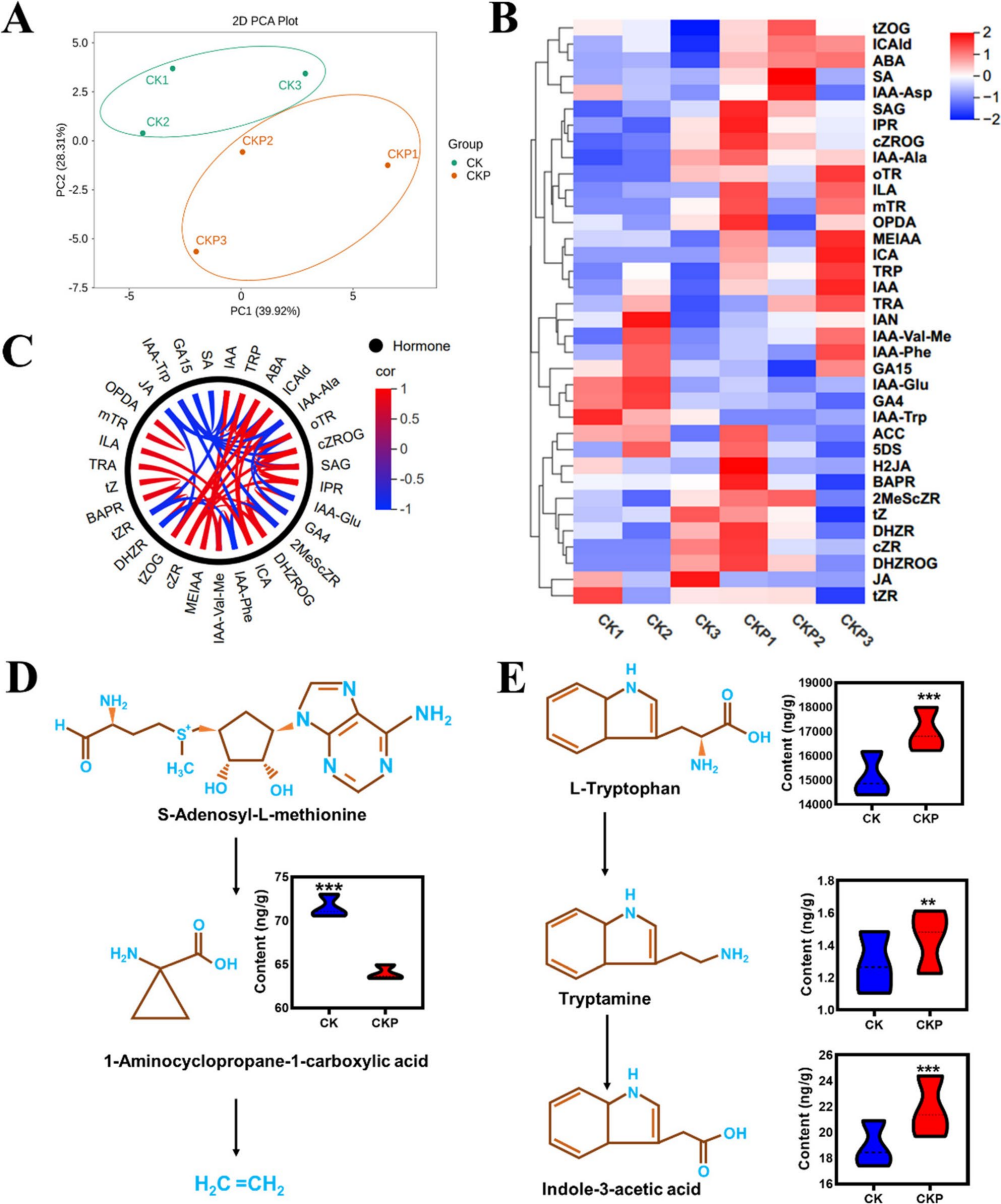
Key differentially expressed hormones screening and synthetic pathway analysis. Note: (**A**) Differential metabolite sample PCA plot; (**B**): Differential plant hormone heatmap; (**C**): Differential plant hormone correlation diagram; (**D**): Ethylene synthesis pathway; (**E**): Auxin synthesis pathway

We detected 42 plant hormones across two experimental groups for targeted plant hormone omics analysis. PCA analysis clearly distinguished the two groups, indicating that plant hormone differences in this experiment were induced by a single variable, strain UW4 (Fig. 6A). All identified plant hormones in the heatmap were categorized into eight groups, including abscisic acid (1), auxin (14), cytokinin (12), ethylene (1), gibberellin (2), jasmonic acid (3), salicylic acid (2), and Strigolactone (1). Strain UW4 significantly increased tZOG (trans-Zeatin-O-glucoside), ICAld (Indole-3-carboxaldehyde), TRP (L-tryptophan), TRA (Tryptamine), IAA (Indole-3-acetic acid), and ABA (Abscisic acid) levels while markedly reducing the ACC (1-Aminocyclopropanecarboxylic acid) content. Strain UW4 also promoted mTR (meta-Topolin riboside) accumulation, thereby enhancing cell division and plant growth. Correlation analysis further elucidated the mutual regulatory relationships among differentially expressed plant hormones: IAA, TRP, ICAld, IAA-Ala (N-(3-Indolylacetyl)-L-alanine), Tzog (trans-Zeatin-O-glucoside), cZR (cis-Zeatin riboside), and DHZROG (Dihydrozeatin-O-glucoside riboside) showed positive correlations, while ABA exhibited negative correlations with tZR (trans-Zeatin riboside), JA (Jasmonic acid), and IAATrp (Indole-3-acetyl-L-tryptophan) (Fig. 6B-C).

Combined with analysis of key enriched pathways in the transcriptome (Fig. 4), strain UW4 inhibited the expression of ethylene synthesis genes and promoted the expression of auxin signaling pathway genes in garlic. Analysis of compounds in the ethylene and auxin synthesis pathways revealed that strain UW4 reduced 1-aminocyclopropane-1-carboxylic acid content while increasing L-tryptophan, tryptamine, and indole-3-acetic acid (IAA) levels (Fig. 6D-E). This indicates that strain UW4 accelerates growth by decreasing ethylene content and increasing the auxin content in garlic.

### Morphological characteristics and transcriptome correlation analysis

**Fig. 7:**
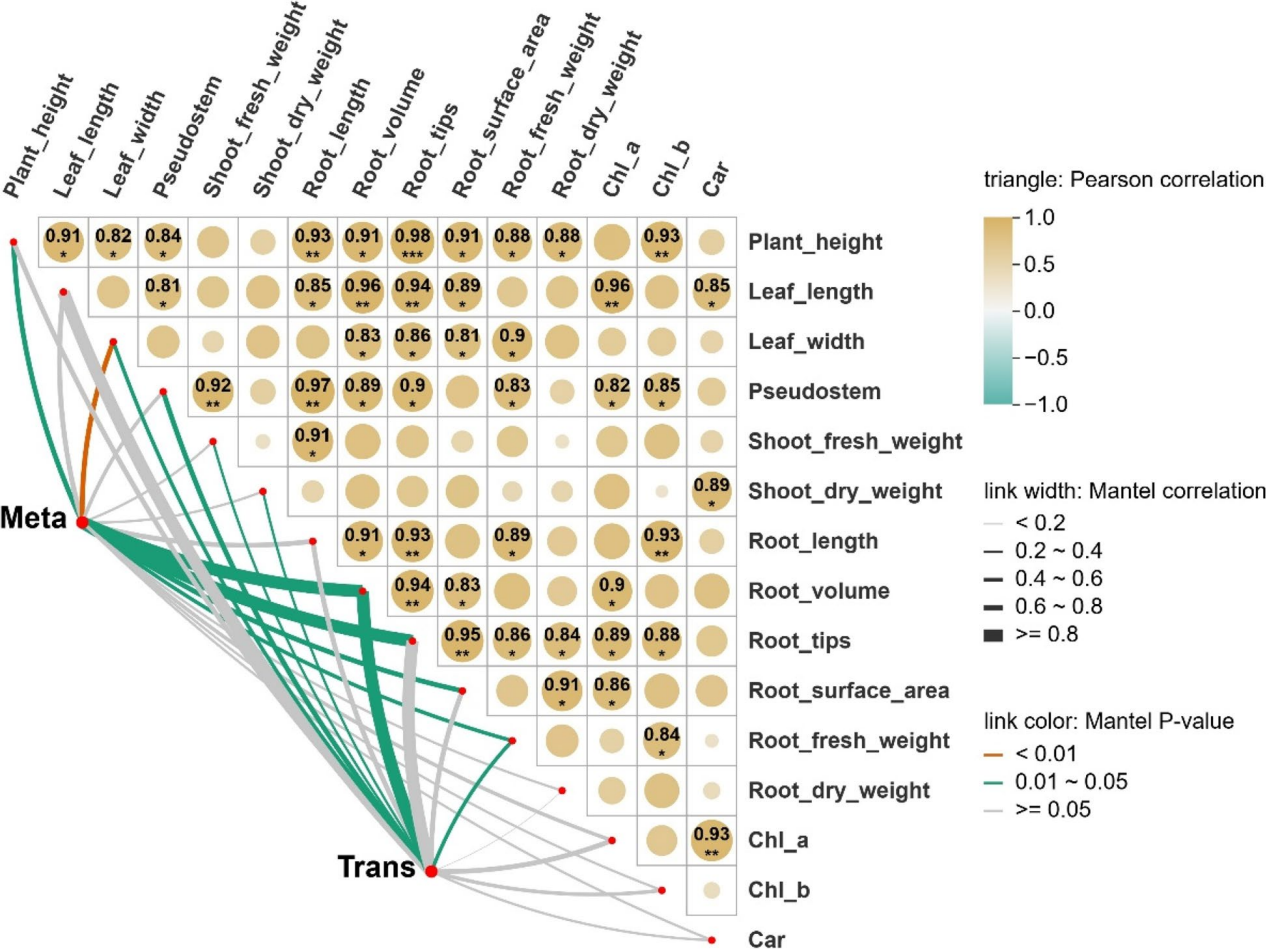
Critical morphological indicators WGCNA analysis. Note: Differential plant hormones, differentially expressed genes and key growth indicators analysed by Mantel test

Correlation analysis revealed a significant positive correlation among garlic growth traits (Fig. 7). Root architectural indicators, including root length, root tip number, and root surface area, showed strong positive interrelationships, indicating coordinated development of the root system. Above-ground traits, such as plant height, were closely associated with shoot fresh weight and dry weight, reflecting the synchronous growth of above-ground parts. Photosynthetic pigment content, including chlorophyll and carotenoids, was positively correlated with both root and above-ground growth traits, confirming the close link between photosynthetic capacity and plant growth. Mantel test results demonstrated that Meta (hormone-related data) and Trans (transcriptomic differential gene data) were closely associated with root development. Meta, representing key phytohormone data, and Trans, representing transcriptional differential genes, both showed highly significant positive correlations with root architectural traits, indicating that hormone metabolism and transcriptional regulation are core factors driving root development. In contrast, their correlations with above-ground growth traits were relatively weak (Fig. 7).

### Plant hormone and transcriptome association analysis

**Fig. 8:**
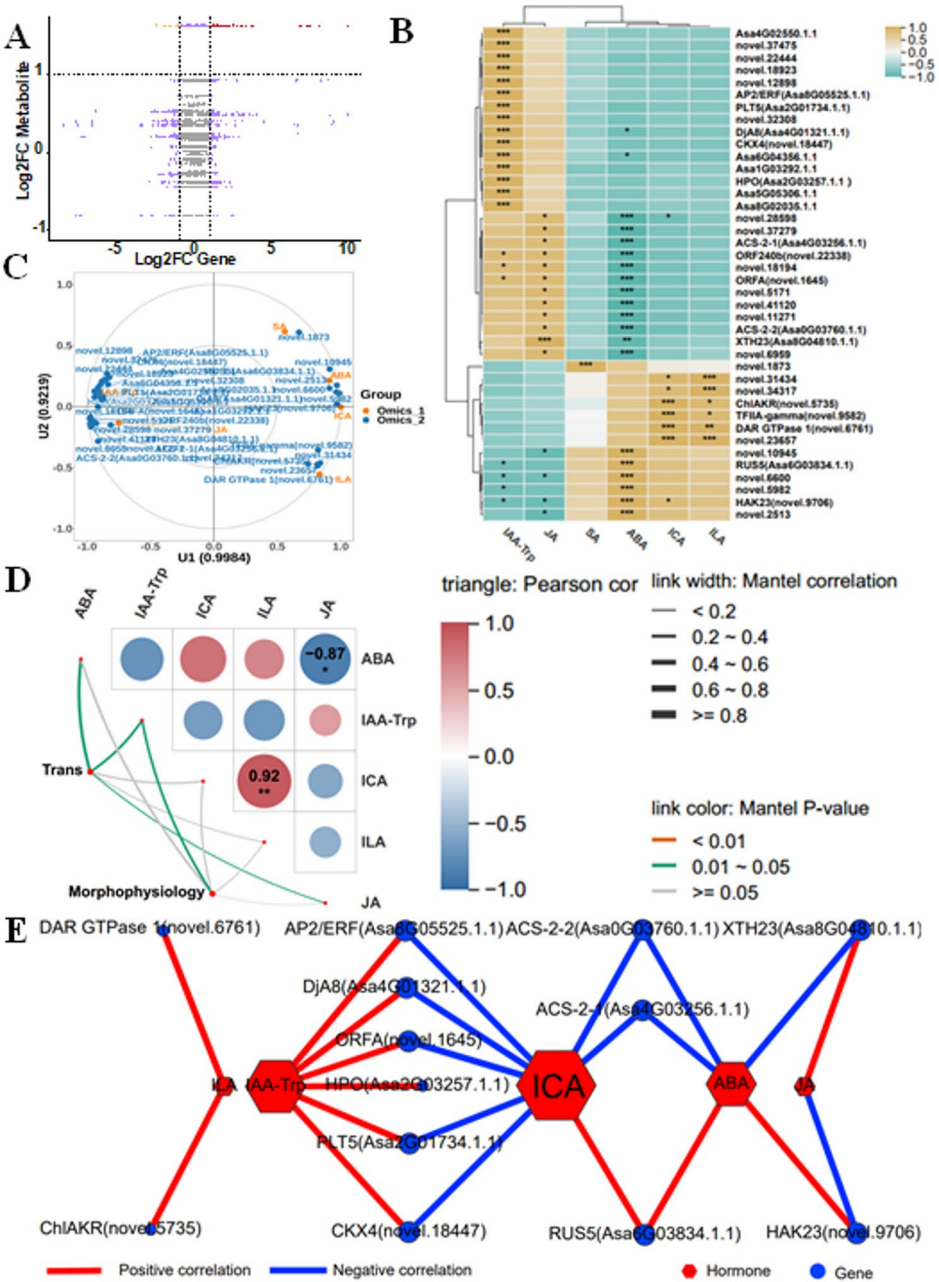
Critical differential plant hormone and transcriptome association analysis. Note: (**A**) Nine-quadrant co-expression diagram of differentially expressed plant hormones and genes; (**B**) Heatmap of correlations between differentially expressed plant hormones and genes; (**C**) Co-expression association analysis of differentially expressed plant hormones and genes; (**D**) Mantel test analysis of key differentially expressed plant hormones, genes, and morphological indicators; (**E**) Key differentially expressed plant hormones and genes regulatory network diagram

Nine quadrants are delineated by two dashed lines (Log2 FC = ± 1) in Fig. 8-A to distinguish “synergistic/antagonistic” relationships between gene and metabolite changes. Red points (high Log2 FC regions) cluster in the first quadrant (significantly upregulated genes and significantly elevated metabolites), indicating positive synergistic regulation between these genes and metabolites. Combining Fig. 8B and C, genes were grouped into three major functional modules via row clustering. Auxin (IAA-Trp)-specific response genes *AP2/ERF* (Asa8G05525.1.1), Asa4G02550.1.1, novel.37,475, novel.22,444 (top cluster) showed strong positive correlations with IAA-Trp and strong negative correlations with JA, SA, and ABA. Abscisic acid (ABA) specific response genes *ACS-2-1* (Asa4G03256.1.1), *ORF240b* (novel.22338), *XTH23* (Asa8G04810.1.1). Middle cluster showed strong positive correlations with ABA. Salicylic acid (SA) and auxin metabolites (ICA, ILA) response genes novel.31,434, *CHIAKR* (novel.5735), *DAR GTPase1* (novel.6761), *RUS5* (Asa6G03834.1.1) showed positive correlations with SA, ICA, and ILA. Heatmap grouped hormones into two major regulatory clusters via column clustering: auxin and its metabolites (IAA-Trp, ICA, ILA), which jointly regulate numerous positively correlated genes, indicating high synergy in metabolic pathways and signal transduction. Stress-related hormones (JA, SA, ABA) clustered together, exhibiting extensive co-regulation of their target genes, reflecting the mechanism of multi-hormone integration in plant growth responses. Genes such as *ACS* and *AP2/ERF* family members serve as core nodes in multi-hormone crosstalk, potentially acting as molecular switches integrating growth and stress signals in plants. These findings warrant further functional validation.

### Strain UW4 significantly downregulates *1-aminocyclopropane-1-carboxylate oxidase* gene (*ACO*) expression

**Fig. 9:**
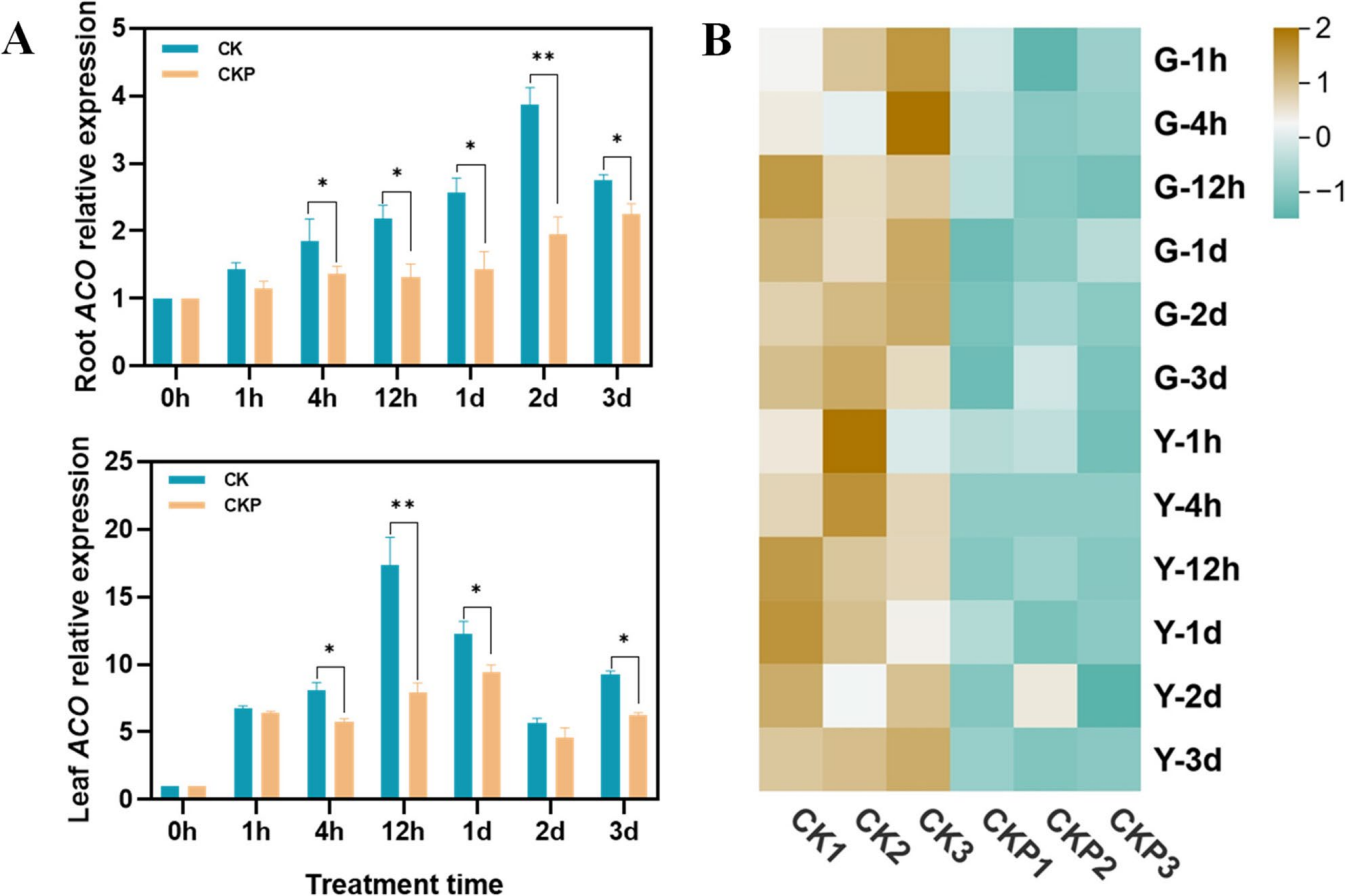
1-aminocyclopropane-1-carboxylate oxidase gene (ACO) expression. Note: (**A**) Relative expression levels of the ACO gene in garlic roots (upper panel) and leaves (lower panel) across different treatment time points (0 h, 1 h, 4 h, 12 h, 1 d, 2 d, 3 d). CK represents the untreated control, and CKP represents the UW4-inoculated treatment group. Data are presented as means and standard error. Asterisks denote significant differences between the CK and CKP groups, with *(*P* < 0.05) and **(*P* < 0.01) (t-test). **B** Heatmap visualization of ACO gene expression patterns in garlic root (G) and leaf (Y) tissues at different time points. CK1-CK3 denote three biological replicates of the control group, and CKP1-CKP3 denote three biological replicates of the UW4-inoculated group. The color scale indicates normalized expression levels (Z-score), with brown representing high expression and cyan representing low expression

Combined qRT‑PCR quantification (Fig. 9A) and expression heatmap analysis (Fig. 9B) systematically revealed the regulatory effects of UW4 on transcript levels of *ACO*, a key rate-limiting gene in ethylene biosynthesis, in garlic roots and leaves. Under control conditions (CK), *ACO* displayed distinct time-dependent basal expression: transcript levels increased steadily in roots and peaked at 2 d, while in leaves they rose rapidly to a sharp maximum at 12 h, with overall abundance markedly higher than in roots. Inoculation with UW4 (CKP) significantly repressed *ACO* expression at all time points in both tissues, with the strongest inhibition occurring at 2 d in roots and 12 h in leaves. Heatmap results further confirmed globally lower *ACO* transcript levels in the CKP group than in the CK group, with high stability across biological replicates. These data demonstrate that UW4 reduces ethylene production by transcriptionally downregulating *ACO* in garlic, providing direct molecular evidence for its growth-promoting role.

## Discussion

*Pseudomonas* is one of the most diverse and prevalent genera found throughout all natural environments. *Pseudomonas* sp. UW4 is a well-studied PGPB isolated from the rhizosphere of reeds growing on the campus of the university of Waterloo in Ontario, Canada [33]. Recent research indicates that the PGPB *Pseudomonas* sp UW4 coordinates ACC deaminase and trehalose to synergistically protect tomato plants from salt stress [34]; ACC deaminase from strain UW4 protects several plants from salt stress including canola [35], cucumber [36], and barley [37]. Concurrently, PGPB inoculation significantly enhances the synthesis and accumulation of proline and soluble sugars within plants under salt stress, maintaining cellular water potential and osmotic balance. Direct observations confirm its positive effects on plant physiological indicators [38, 39]. Strain UW4 inoculation significantly increased garlic root length, surface area, root tip number, and root volume. This finding aligns perfectly with results from studies on peppers [40], consist with the universality of PGPB that contain ACC deaminase and IAA in promoting root morphogenesis. Crucially, root tip number strongly suggests that UW4 influences root apical meristem activity by regulating endogenous hormones. This robust root architecture directly expanded the interface for water and nutrient uptake, providing a solid physical foundation and physiological support for the remarkable accumulation of aboveground biomass observed [41]. Strain UW4 treatment also increased chlorophyll a, b, and carotenoid content in garlic leaves, signifying a qualitative leap in light energy capture capacity. This phenomenon echoes reports of SynCom inoculation promoting chlorophyll synthesis in peppers, but with more pronounced effects [42]. Enhanced photosynthetic performance extends beyond pigment accumulation and likely involves upregulation of photosystem II core protein genes, thereby improving photosystem stability and efficiency. Strain UW4 might systemically regulate host carbon and nitrogen metabolism, like *Stenotrophomonas pavanii* KC98 activates soluble sugar synthesis pathways [41], while *Bacillus megaterium* NCT-2 drives nitrogen cycling and induces amino acid metabolism [43]. Strain UW4 jointly promotes garlic growth and yield formation by optimizing root architecture to enhance absorption capacity and improving photosynthetic performance to boost assimilation capacity.

Many PGPB strains regulate endogenous auxin metabolism and signal transduction through multiple mechanisms, directly influencing plant height, root architecture, and biomass accumulation. *Sinomonas gamaensis* NEAU-HV1 directly regulates auxin signaling by reconfiguring the IAA14-ARF7/19 transcription factor interaction network, thereby promoting lateral root development [44]. Potato plants specifically recruit *Pantoea* sp. MCC16, high-IAA-producing bacterium secreting naringin, forming a positive feedback loop [45]. These findings reveal the complexity and diversity of auxin regulatory networks in PGPB-plant interactions. Regarding auxin metabolism, strain UW4 treatment significantly increased the content of IAA synthesis precursors such as tryptophan (TRP) and tryptamine (TRA), promoting endogenous IAA biosynthesis. This mechanism of activating the host’s own synthetic system contrasts sharply with the direct IAA secretion pathway of *Myrmecridium schulzeri* B-4 [46]. At the molecular level, UW4 specifically induced upregulation of *SAUR* family genes, promoting cell wall acidification by activating plasma membrane H⁺-ATPase. This finding complements the mechanism by which *Bacillus subtilis* WM13-24 regulates auxin polar transport via volatile organic compounds, jointly facilitating cell expansion growth [47]. Regarding ethylene metabolic regulation, strain UW4 significantly reduced both ACC content and ethylene biosynthesis rates by suppressing key ethylene synthesis genes such as *ACS*. This upstream regulatory strategy shares a similar effect with strains such as *Bacillus subtilis* JPVS11, which degrades ACC by producing ACC deaminase [48]. Notably, strain UW4’s synergistic regulation of auxin and ethylene signaling pathways represents a more systematic approach compared to the single-point intervention of IAA14-ARF7/19 protein interactions by *Sinomonas gamaensis* NEAU-HV1 [44], potentially explaining UW4’s more pronounced growth-promoting effects. Compared to many other PGPB strains reported in the literature, strain UW4 exhibits distinct mechanisms of action [49] *Stenotrophomonas pavanii* KC98 primarily influences plant hormones indirectly by shaping specific rhizosphere microbial communities [50], while *Streptomyces lasalocidi* JCM 3373ᵀ remotely regulates root architecture by secreting indole-3-carboxaldehyde (ICAld) [51]. In contrast, strain UW4 directly colonizes the rhizosphere and regulates host hormone metabolism and signaling through multiple pathways. This direct mechanism likely contributes to its more pronounced growth-promoting effects. At the level of hormone interaction networks, strain UW4-induced hormonal changes reveal a growth-promoting module positively correlated with auxin and its metabolites, while stress-related hormones like ABA and JA form a defense module are negatively correlated with growth. This coordinated regulatory pattern echoes the hormonal changes observed in *Codonopsis pilosula* by *Serratia fonticola* CPSE11 [52], though strain UW4 exhibits a more refined regulatory network. Notably, *AP2/ERF* family transcription factors serve as core nodes integrating multiple hormone signals. *Halomonas alkaliantarcticae* M23 (M23) enhances antioxidant levels and regulates abscisic acid (ABA) levels in maize [53], while strain UW4 demonstrates broader regulatory dimensions through simultaneous modulation of multiple hormone pathways. While increasing endogenous ABA content, strain UW4 reduces signal sensitivity by suppressing *PYL* receptor gene expression. This sophisticated regulatory strategy allows plants to maintain baseline stress resistance while mitigating ABA’s growth inhibition, similar to the ABA-elevating mechanism of *Bacillus polymyxa* HL14-3 [54]. Similar to *Bacillus cereus* mitigating drought stress primarily through ACC deaminase and auxin pathways, strain UW4 refines its growth-promoting network by synergistically regulating multiple auxin, ethylene, and ABA pathways. This multi-target regulatory characteristic likely underpins strain UW4’s consistent high-efficiency growth promotion across diverse environmental conditions. A clear causal relationship exists between the hormone remodeling induced by strain UW4 and the observed phenotypic changes. This organ-specific hormonal control parallels the mechanism by which *Bacillus subtilis* sp. 5B1 mediates root tropic growth through root-crown-specific regulation of PIN proteins, though strain UW4 exhibits broader regulatory scope [55].

*Pseudomonas* sp. UW4 exerts a significant growth-promoting effect on garlic through multiple mechanisms, including optimizing root system architecture to enhance nutrient uptake, increasing the accumulation of photosynthetic pigments and improving photosynthetic efficiency, as well as systemically reprogramming the host plant’s hormone network. This synergistic regulatory effect primarily facilitates biomass accumulation by enhancing auxin signaling, suppressing ethylene biosynthesis, and precisely modulating abscisic acid sensitivity. Strain UW4 offers a robust theoretical basis and valuable microbial resources for the development of next-generation agricultural inoculants. Future research should prioritize verifying its efficacy and persistence under diverse field scenarios. Given its unique hormonal regulatory mechanism, exploring the potential of constructing synergistic microbial consortia may yield more stable and potent growth-promoting effects.

## Data Availability

The datasets generated during the current study are available in the NCBI repository, [PRJNA1405895].
